# Poor Recognition of Risk Factors for Hepatitis B by Physicians Prescribing Immunosuppressive Therapy: A Call for Universal Rather than Risk-Based Screening

**DOI:** 10.1371/journal.pone.0120749

**Published:** 2015-04-15

**Authors:** Alissa Visram, Kelvin K. W. Chan, Phyllis McGee, Jordana Boro, Lisa K. Hicks, Jordan J. Feld

**Affiliations:** 1 Toronto Centre for Liver Disease, University Health Network, University of Toronto, Toronto, Canada; 2 St. Michael’s Hospital, University of Toronto, Toronto, Canada; 3 Sunnybrook Odette Cancer Centre, University of Toronto, Toronto, Canada; 4 University Health Network, Toronto, Canada; CRCL-INSERM, FRANCE

## Abstract

**Background:**

Reactivation of hepatitis B virus (HBV) during immunosuppressive therapy (IST) can lead to severe and even fatal hepatitis but can be largely prevented with prophylactic antiviral therapy. Screening for HBV prior to starting IST is recommended. Both risk-based and universal screening have been recommended by different societies. For effective risk-based screening, physicians must be aware of risk factors for chronic HBV infection.

**Methods:**

The HBV screening practices prior to starting IST of rheumatologists, medical and hematological oncologists were evaluated by survey and chart review. Country of origin, the primary risk factor for HBV exposure, was determined in all patients.

**Results:**

Of 140 rheumatology, 79 medical oncology and 53 hematology patients reviewed, 81%, 11% and 81% were deemed to be at high risk of HBV reactivation by their physicians respectively, however only 27%, 6% and 62% (p<0.0001) were actually screened for HBV prior to starting IST. For patients from HBV-endemic regions, more hematology patients (53%) were correctly identified by their physicians as being at high risk of reactivation than rheumatology patients (2.4%, p=0.0001) or medical oncology patients (15%, p=0.009). However actual screening rates were not increased in patients from endemic regions. A total of 81 patients were screened for HBsAg; 2 were positive. Of the 33 patients screened for anti-HBc, 10 (30%) were positive.

**Conclusions:**

Hematologists, rheumatologists and medical oncologists had low rates of screening for HBV prior to prescribing IST, largely due to poor identification of those at risk for infection. Risk-based screening strategies are unlikely to be effective and should be replaced by universal screening.

## Introduction

Chronic hepatitis B virus (HBV) infection affects more than 240 million people worldwide with the highest prevalence in East and Southeast Asia and Sub-Saharan Africa [1]. North America has a low burden of HBV infection in the general population, however in large urban centers HBV prevalence may be above 2%[2]. Most chronic HBV infections are acquired very early in life through vertical or early horizontal transmission. Although chronic infection may cause progressive liver injury leading to cirrhosis, liver failure and/or liver cancer, most individuals are initially entirely asymptomatic. Consequently, many individuals are unaware of their HBV infection. For instance, in the largest community study to date, only 35% of HBV-infected Asian Americans were aware of their status[3].

In most adults with chronic HBV, the immune system is able to control viremia; however, natural or iatrogenic immunosuppression can lead to a loss of immune control resulting in reactivation of HBV replication. Clinically HBV reactivation varies from asymptomatic increases in transaminases to fulminant liver failure [4, 5]. In addition to the hepatic consequences, HBV reactivation may lead to an interruption or cessation of planned chemotherapy, potentially compromising cancer outcomes[6]. Fortunately, HBV reactivation can be effectively prevented with prophylactic antiviral therapy [7–11].

The Centers for Disease Control and Prevention (CDC), as well as the European, Asian and American liver societies, all recommend that patients scheduled to receive IST be screened for hepatitis B surface antigen (HBsAg) with institution of antiviral therapy for all positive individuals [11–14]. In contrast, the American Society of Clinical Oncology (ASCO) recommends in a Provisional Clinical Opinion (PCO) that “physicians may consider screening patients belonging to groups at heightened risk for chronic HBV infection or if highly immunosuppressive therapy is planned”[15]. Similar to ASCO, the American College of Rheumatology (ACR) recommends screening of patients for HBV if they have known risk factors for chronic HBV infection, which they list as a history of injection drug use, occupation in a healthcare profession or multiple sexual partners [16, 17].

Screening only high-risk individuals for HBV requires that physicians are able to reliably identify individuals at high risk for HBV infection. We evaluated the screening practices of rheumatologists, medical oncologists and hematologists prior to starting IST and assessed whether they could identify patients at high risk of chronic HBV infection.

## Methods

All medical oncologists, malignant hematologists and rheumatologists working at a single, large, teaching hospital in Toronto, Canada, were invited to participate in the study. Medical oncologists and hematologists were asked about screening for HBV and cardiac dysfunction prior to the use of anthracycline-based chemotherapy. Rheumatologists were asked about screening for HBV, HIV and tuberculosis (TB) prior to the use of biologic disease modifying anti-rheumatic agents (DMARDs) (abatacept, adalimumab, etanercept, infliximab, rituximab) or non-biologic DMARDs (azathioprine, leflunomide, methotrexate, sulfasalazine). The purpose of asking physicians about their screening practices for other diseases was to blind physicians to the focus of the study.

Patients started on an immunosuppressive regimen by the physician completing the survey were approached independently after their scheduled clinic visit, and, if they agreed to participate, their country of origin was documented. Patients were not asked about other risk factors for HBV because the majority of chronic HBV infections result from vertical or early childhood transmission and are thus largely related to the prevalence of HBV in the country of origin. A minimum of five patients per participating-physician were recruited. After each patient’s clinic visit, physicians were asked whether they would screen the patient for each condition, whether the patient was at increased risk for those conditions and if so, which risk factors were present. The physician’s specialty and level of training were recorded. After physicians had completed the patient-specific questionnaires, they were given a second questionnaire pertaining to their general screening practices, their awareness of guidelines about HBV screening, and their choice of HBV screening markers.

A chart review was conducted on all patients for whom the interviewed physician made the decision to start IST. Each patient’s diagnosis, the type and start date of IST, the method of HBV screening (i.e. the specific serologic markers used), and the test outcome were recorded. Clinic notes were reviewed for documentation surrounding the rationale for HBV screening.

Patients originating from countries with intermediate or high rates of chronic HBV infection (prevalence of HBsAg greater than or equal to 2% in the general population) were deemed to be originating from HBV-endemic countries and thus at increased risk of chronic HBV infection. HBV-endemic countries were identified according to the 2008 CDC guidelines on chronic HBV management [16].

### Statistical Analysis

Descriptive statistics were used to report results and where applicable proportions were compared using Fisher’s Exact Test. Continuous variables were compared using the Student’s T test or the Wilcoxon Rank sum test, as appropriate. Analysis was performed using Stata 9.2 (College Station, TX).

### Ethics Statement

The study was approved by the Research Ethics Board of the University Health Network. Study participants provided written informed consent for their participation. They were provided with an explanation of the study, which concluded with the sentence "Your consent to participate in this research study is demonstrated by your voluntary completion of the following questionnaire.” According to our Research Ethics Board policy, completion of study questionnaires by physicians constitutes written informed consent. A record of all participants approached for the study was maintained. For the chart review component of the study, written informed consent was not obtained from patients. All data extracted from patient records were anonymized and de-identified prior to analysis as is required by our Research Ethics Board for chart review studies.

## Results

Of the physicians approached, 16 of 17 rheumatologists (94%), 12 of 18 medical oncologists (67%) and 9 of 17 hematologists (53%) agreed to participate in this study. One rheumatologist, 3 medical oncologists and 3 hematologists were excluded because they had fewer than five patients on IST during the recruitment clinic (see table 1). All 272 patients approached (140 rheumatology, 79 oncology, and 53 hematology patients) agreed to participate. As outlined in table 2, 88 patients were born in regions endemic for HBV; 42 rheumatology patients (30%), 27 oncology patients (34%), and 19 hematology patients (36%). A majority of patients from HBV-endemic areas originated from Asia, although other regions were also well represented (see table 2).

**Table 1:** Demographics of Physician Respondents.

	Rheumatologists	Medical Oncologists and Hematologists
Number of physicians	15	15
Number of patients	140	132
Staff physician	10	15
Clinical Fellow	5	0
Location of training		
North America	9	14
Asia	5	0
Europe	0	1
Number of years in practice		
< 5	3	0
5–10	6	8
11–20	1	6
> 20	5	1
Specialty		
Hematology	-	6
Medical Oncology	-	9
Gender		
Male	11	9
Female	4	6

**Table 2:** Patient Characteristics.

	Rheumatology		Medical Oncology		Hematology	
N—all patients	**140**		**79**		**53**	
N’—patients included in chart review	**73**		**78**		**53**	
All patients from HBV Endemic Region	**27**		**19**		**42**	
Africa	0		2		5	
East Asia	7		4		6	
Southeast Asia	6		5		4	
South Asia	3		4		9	
Eastern Europe	7		1		7	
South America	0		0		8	
Caribbean	4		3		3	
**Treatment Regimen/Primary Diagnosis** of patients included in the chart review	MTX	18 (25%)	GI	34 (44%)	Lymphoma	27 (51%)
	Non biologic DMARD	8 (11%)	Breast	34 (44%)	Leukemia	26 (49%)
	Biologic DMARD	14 (19%)	Sarcoma	10 (12%)		
	MTX + DMARD	5 (7%)				
	MTX + Biologic	28 (38%)				

### Physician ability to identify patients at risk of chronic HBV infection

Overall, physicians identified 38 patients as having risk factors for chronic HBV infection. Of those patients, 55% (n = 21) were identified to have risk factors that would most likely lead to acute HBV with clearance of HBsAg in most adults (prior injection drug use, sexual exposure, blood transfusion, and travel to endemic areas). Rheumatologists identified 7 of 140 (5%) patients as having risk factors for HBV infection; medical oncologists identified 9 of 79 (11%) of patients as having risk factors for chronic HBV infection and hematologists identified 22 of 53 (32%) patients as having risk factors for chronic HBV infection. Hematologists and medical oncologists most commonly identified origin from an HBV-endemic area as a risk factor for chronic HBV infection, while rheumatologists tended to identify renal transplant recipients or healthcare workers as being at risk of chronic HBV infection.

**Fig 1:**
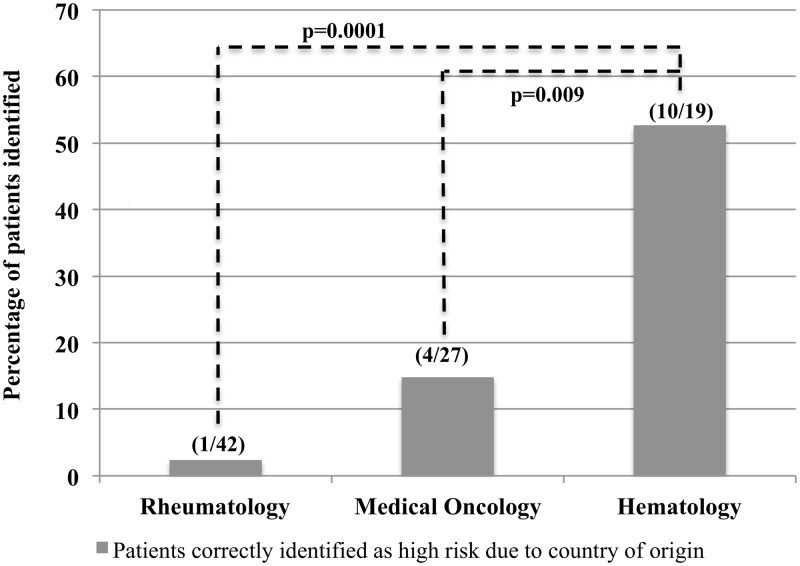
Physician ability to identify patients at high risk of chronic HBV infection based on country of origin. Patients originating from areas with greater than an intermediate rate (≥2) of chronic HBV infection, as determined by the CDC [16], were identified through the patient questionnaire. This figure illustrates the proportion of these patients who were correctly identified by their treating physician as being at high risk of HBV reactivation due to their country of origin.

Of the 88 patients born in HBV-endemic regions, country of origin was cited as a risk factor in only 15 (17%) (see Fig 1). Patients from East Asia and Southeast Asia were more commonly identified as being at high risk for HBV infection (East Asia 5 of 17 and Southeast Asia 5 of 15), whereas only 2 of 16 from South Asia, 1 of 15 from Eastern Europe, 1 of 7 from Sub-Saharan Africa, 1 of 10 from the Caribbean, and 0 of 8 patients from endemic areas within South America were identified as being at risk for HBV.

Only 1 of the 42 (2.4%) rheumatology patients from an HBV-endemic region (Guyana) was identified as being at increased risk for HBV. In contrast, 7 of the 22 (32%) patients originating from areas with a high prevalence of TB were identified as being at risk of latent TB infection.

Among medical oncologists, 4 of the 27 (15%) patients from HBV-endemic areas were identified as being at increased risk of HBV infection. Only 2 of the 9 medical oncologists correctly recognized all of their patients from HBV-endemic areas as being at increased risk of HBV, whereas 6 of 9 did not identify any patients born in HBV-endemic regions as being at high risk.

Finally, hematologists recognized that 10 of the 19 (53%) patients from HBV-endemic countries were at increased risk of chronic HBV infection, but only 2 of the 6 hematologists correctly recognized that all of their patients from HBV-endemic regions were at increased risk. Hematologists were significantly more likely to identify patients from HBV-endemic areas compared to medical oncologists (p = 0.009) and rheumatologists (p<0.001).

No associations were observed between physician ability to correctly identify patients at risk of chronic HBV and physician-gender, location of medical training (North America v. Asia), or duration of medical practice (data not shown).

### Physicians’ actual HBV screening patterns

Rheumatologists indicated through the questionnaires that 113 of the 140 patients should receive screening for chronic HBV prior to IST. Only 73 of the 140 rheumatology patients were started on IST by the physician in the study and were thus included in the chart review. Of these 73, 65 (89%) were identified in the survey as meriting HBV screening by their physician. However, only 19 (26%) had documentation of HBV serology prior to starting IST. One additional patient who was not identified as being at high risk of HBV infection was screened before treatment, bringing the total screening rate to 20 of 73 (27%). In contrast, of the 19 patients (26%) treated with biologics who were identified as being at increased risk of TB, 17 (90%) had documented TB skin tests before starting therapy (p<0.0001). There was no difference in the rate of HBV screening between patients treated with methotrexate (n = 51) and those who received biologic DMARDs (n = 42) (methotrexate: 11 of 51 (22%) vs. biologics: 13 of 42 (31%), p = 0.35). In the 28 patients who received both treatments, 4 were screened for HBV prior to starting methotrexate and 5 were screened after starting methotrexate before going on a biologic, while 19 (68%) had no screening. In addition to the patients who were screened before starting IST, 9 patients (12%) had HBV screening performed for reasons unrelated to, and after the start of, immunosuppressive medications. The reasons for screening were routine prenatal screening (n = 1), work-up for elevation of transaminases (4), diagnosis of hepatitis C virus infection (n = 1), planned renal transplant (n = 1), work-up of a fever of unknown origin (n = 1) and no clearly documented reason (n = 1).

Similarly, screening for HBV in medical oncology and hematology patient populations was inconsistent prior to immunosuppression. Collectively, medical oncologists indicated through the questionnaires that only 9 of the 78 (12%) patients evaluated would warrant HBV screening. Chart review confirmed HBV screening in only 3 of these 9 patients prior to chemotherapy, as well as 2 additional patients who were not identified as being at high risk by their physicians, bringing the total screening rate to 5 of 78 (6%). In contrast, medical oncologists reported that 33 of 34 (97%) patients treated with anthracycline chemotherapeutic agents should have cardiac testing prior to chemotherapy (p<0.0001). In addition to the 5 patients (6%) who were screened prior to receiving chemotherapy, 7 patients had HBV serology performed after the initiation of chemotherapy: 4 for elevated transaminases, 1 for post-operative jaundice, and 2 as part of a clinical trial protocol.

Hematologists responded in their questionnaires that 43 of 53 (81%) patients seen would require HBV screening. Review of these 43 patient charts showed that 31 (72%) were actually screened for HBV prior to chemotherapy. Two patients who were not identified as requiring HBV screening also had HBV serology performed bringing the total to 33 of 53 (62%) hematology patients. An additional 7 patients had HBV serology performed after starting chemotherapy: 6 prior to bone marrow transplantation and 1 as part of the investigation of elevated transaminases. Hematologists identified 32 of 39 (82%) patients requiring screening for cardiac dysfunction prior to anthracycline-based chemotherapy (p = 0.06). A summary of the above findings is presented in table 3.

**Table 3:** Summary of Screening Patterns.

	Total number of patients		Number of patients from HBV-endemic areas		Number of patients identified by physicians as needing screening or actually screened prior to IST		Number of patients from HBV-endemic areas identified as needing screening or actually screened prior to IST	
	Survey	Chart review	Survey	Chart review	Identified as high risk	Actually screened	Identified as high risk	Actually screened
Rheumatologist	140	73	42	22	113 (81%)	20 (27%)	29 (69%)	4 (18%)
Medical Oncologist	79	78	27	27	9 (11%)	5 (6%)	5 (19%)	2 (7%)
Hematologist	53	53	19	19	43 (81%)	33 (62%)	16 (84%)	12 (63%)

IST—immunosuppressive therapy

HBV screening prior to IST was significantly higher among hematologists (33 of 53 patients, 62%) compared to medical oncologists (5 of 78 patients, 6%; p = 0.0001) and to rheumatologists (20 of 73 patients, 27%; p = 0.0001). Medical oncologists were also significantly less likely to screen patients prior to IST when compared to rheumatologists (p = 0.001).

### Screening of patients from HBV-endemic areas

**Fig 2:**
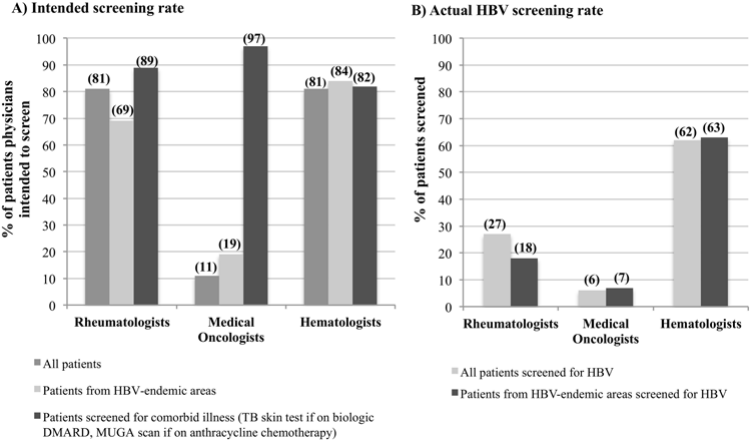
Screening practices of physicians prior to immunosuppressive therapy. A) The proportion of patients identified to merit screening for HBV prior to immunosuppressive therapy in each specialty are shown. This is contrasted with the proportion of patients requiring TB screening before biologic DMARDs (rheumatology) and cardiac testing prior to anthracycline-based chemotherapy (hematologists/oncologists). B) The rates of planned screening are contrasted with the actual rate of HBV screening for HBV prior to immunosuppressive therapy for all patients and for the sub-group of patients from HBV-endemic countries.

In order to assess whether physicians selectively screened patients based on their risk factors, the screening rate of patients from HBV-endemic areas was also considered. Of the 22 rheumatology patients included in the chart review from HBV-endemic areas, only 4 (18%) were screened for HBV prior to starting treatment. Similarly 2 of the 27 (7%) medical oncology patients from HBV-endemic areas were screened before chemotherapy. Of the 53 hematology patients included in the chart review, 19 were from HBV-endemic countries and 12 (63%) were screened before chemotherapy. For the remainder of patients originating from HBV-endemic areas, physicians rationalized their decision not to screen by reporting that patients were at low risk for HBV infection. Therefore, the screening rates among patients from HBV-endemic regions was similar to the overall screening rates in each group, suggesting that physicians were not selectively screening patients at high risk of chronic HBV infection (Table 3 and Fig 2).

### Choice of screening test used to identify patients at risk chronic HBV

Only 6 of 27 physicians reported that they would screen for all 3 HBV serological tests (HBsAg, hepatitis B surface antibody (anti-HBs), and core antibody (anti-Hbc). Most physicians stated that they would screen using HBsAg and anti-HBs. Eight of 27 physicians reported that they would screen for HBV using liver enzymes. Other tests performed included anti-HBc IgM, hepatitis B e Ag (HBeAg) and anti-HBe as well as HBV DNA in 2 patients, both of whom were HBsAg positive. According to the chart review, all patients screened by hematologists were screened for HBsAg, 81% had anti-HBs testing and 54% were screened for anti-HBc. In contrast, although 93% of rheumatologists stated that they would use HBsAg as a screening test, only 79% of their screened patients had this test performed and only 25% had testing for anti-HBc. Of the 81 patients screened for HBV, 9 (11%) were screened after starting IST due to elevated liver enzymes.

In total, 33 of the 204 patients included in the chart review were screened for anti-HBc. Anti-HBc testing was done primarily by hematologists, accounting for 21 of the 33 (64%) patients tested. Of these 33 patients, 10 (32%) were anti-HBc positive, 4 (40%) of whom were born in countries with an intermediate to high prevalence of HBV infection, as defined by the CDC. Using the more inclusive list of HBV-endemic countries defined by the AASLD, 6 (60%) of patients who tested positive for anti-HBc originated from an “endemic area”.

Of the 81 patients who had any HBV serology performed, 74 were tested for HBsAg, and 2 (3%) patients were positive. Both HBsAg positive patients were already known to have chronic HBV before the decision to initiate IST, and were followed by hepatologists. Both were from HBV-endemic countries. Both were treated with lamivudine prophylaxis prior to starting chemotherapy and neither patient experienced HBV reactivation.

## Discussion

Three possible approaches to screening for HBV prior to IST exist: no screening, selective screening of patients at high-risk of chronic HBV infection, and universal screening of all patients scheduled to receive immunosuppression. Given the potentially grave consequences of HBV reactivation and the availability of effective antiviral prophylaxis, most groups advocate for some type of HBV screening. ASCO and ACR both recommend selective screening of high-risk individuals; the CDC and AASLD recommend universal screening (as summarized in Table 4) and the US Preventative Services Task Force (USPSTF) cites the CDC recommendation but gives no specific guidance on screening of patients prior to immunosuppression [18].

**Table 4:** Summary of current guidelines regarding HBV screening.

	ACR [16, 17]	CDC[13]	ASCO[15]	AASLD[12]
**HBV screening recommended for:**	Patients with HBV risk factors who are scheduled to receive methotrexate or leflunomide.	All patients receiving cytotoxic or immunosuppressive therapy	Patients at high-risk of chronic HBV, or if highly immunosuppressive therapy is planned.	All patients requiring immunosuppressive therapy
**Recommended screening tests**	Tests “might” include: HBsAg, Hepatitis B antibodies, Anti-HBc	HBsAg, Anti-HBc, Anti-HBs	HBsAg Anti-HBc (in some, populations)	HBsAg and Anti-HBs OR Anti-HBc*

*As per the AASLD guidelines, patients can be tested with anti-HBc alone provided that patients who test positive are further tested for HBsAg and anti-HBs to differentiate infection from immunity.

For a selective screening strategy to be effective, physicians who administer IST must be able to reliably identify patients at high risk of chronic infection. Our study suggests that this prerequisite may be a problem in rheumatology, oncology and hematology clinics. Physicians identified only 17% of patients from HBV-endemic areas as being at risk of chronic HBV infection. Hematologists were significantly better able to identify high-risk patients than rheumatologists and medical oncologists, however, even hematologists failed to identify 47% of their patients from HBV-endemic areas as being at high risk of chronic HBV infection. Although the ACR and ASCO guidelines call for selective screening of high-risk patients prior to the initiation of IST, only 18 of 68 (26%) patients originating from HBV-endemic countries were screened for HBV prior to starting IST.

Physicians were more likely to identify patients from East and Southeast Asia as being at high-risk for chronic HBV infection; patients from endemic regions in Sub-Saharan Africa and South America were often overlooked. This is concerning because 5–10% of the adult population in Sub-Saharan Africa is chronically infected with HBV[1]. These results suggest that risk factor-based screening would likely miss many high-risk individuals.

Inconsistent and imprecise recommendations may contribute to suboptimal screening. As outlined in table 4, professional medical societies have made markedly different recommendations and none clearly defines who is a high-risk patient. For instance, the ASCO provisional opinion on HBV does not specify what factors put a patient at high risk for HBV. The ACR guidelines provide some examples of risk factors; however, they omit the most significant risk factor for chronic infection: country of origin. Further complicating matters, the CDC and AASLD guidelines, both of which advocate for universal screening prior to IST, list different countries as having a high prevalence of chronic HBV infection and the USPSTF guidelines on HBV screening cite the CDC recommendations but then do not specifically discuss what to do for patients receiving IST.

Rheumatologists screened 27% of their general patient population for HBV, but only screened 18% of their patients from HBV-endemic regions. The ACR guidelines list injection drug use and occupational and sexual exposure as risk factors for HBV infection. However, these are primarily risk factors for acute rather than chronic infection. In the vast majority (>95%) of immunocompetent adults, HBV exposure results in acute self-limited HBV that does not progress to chronicity[13]. Immunocompetent patients clear HBsAg and remain positive for anti-HBc and generally, anti-HBs, for life. The risk of HBV reactivation with standard IST in individuals who are HBsAg-negative but anti-HBc positive is low and only becomes clinically relevant in those who receive more intense immunosuppression such as stem cell transplantation or anti-CD20 therapy. In contrast, the risk of reactivation is much higher in patients who are HBsAg positive. As the vast majority of such individuals acquired infection either perinatally or in early childhood [19], an individual’s country of origin is the most important risk factor for chronic HBV infection and should be highlighted if selective screening is to be advocated.

Of those rheumatology patients who were at high risk of chronic HBV infection due to their country of origin, only 3 of 11(29%) were screened prior to the initiation of biologic therapy. The ACR guideline is confusing regarding biologics. Screening prior to biologic therapy is not specifically recommended but the use of biologics is contraindicated in patients with untreated chronic HBV infection. Clearly screening is required to ensure that chronic HBV infection is not missed. In the largest review of reported cases, HBV reactivation following anti-TNF therapy occurred in 39% (35/89) of HBsAg positive individuals, including 4 fatal cases of acute liver failure[20], and in 5% (9/168) of HBsAg-negative and anti-HBc-positive individuals, including 1 fatal case. Furthermore, the product monographs for infliximab, etanercept and adalimumab all recommend screening for HBV prior to starting treatment. However, even these recommendations are inconsistent[21–23]. The infliximab product monograph recommends screening all patients for HBV infection; the etanercept and adalimumab product monographs recommend screening only high-risk patients but do not define high-risk.

In our chart review, hematologists had the highest rates of HBV screening. This is likely due to the higher risk of HBV reactivation associated with the more intensive regimens used in this population and the larger body of literature describing the risk of HBV reactivation [24]. Medical oncologists had the lowest rate of reported and actual screening, a trend that has been seen in other studies[25–27]. Day *et al*. reported that oncologists cite anecdotal experience with HBV reactivation as the most common reason for screening patients[27]. Medical oncologists may underestimate the risk because few have seen cases of HBV reactivation in their practice[25, 26]. As well, the risk of HBV reactivation with solid tumors is poorly documented and may be less broadly appreciated in the oncology community.

The true prevalence of HBV in this study could not be determined due to the low screening rate. Of those patients screened for HBsAg, 2 of 74 (2.7%) were positive. Of those patients screened for anti-HBc, 10 of 33 (30%) were positive. Of these 10 patients, 4 were from HBV-endemic areas as defined by the CDC and 6 were from HBV-endemic areas as defined by the AASLD. This suggests that, in large urban centres with a large foreign-born population, a high rate of exposure to HBV may be common even in low prevalence countries such as Canada. More importantly, a significant proportion of positive individuals may be missed if only patients from HBV-endemic areas are screened.

Universal screening guidelines would be easier to implement than selective screening of high-risk patients and would leave minimal room for misinterpretation. The major argument against universal screening is that in low prevalence countries the yield and therefore cost-effectiveness of this approach may be low. A study by Day and colleagues found that universal screening for HBsAg prior to adjuvant chemotherapy for breast cancer was cost-effective [28]. Zurawska and colleagues have also shown that universal screening for HBsAg is not only cost-effective, but actually cost-saving when compared to no screening or screening only high-risk individuals prior to starting chemotherapy for lymphoma [29]. Importantly, an assumption made in both the Day and Zurawska studies was that targeted screening of high-risk individuals was carried out perfectly. Our results documenting low screening rates and poor awareness of risk factors for chronic HBV infection suggest that physicians are unlikely to correctly identify and screen all high-risk patients, further strengthening the argument for universal HBV screening prior to IST.

The current ASCO, ACR, AASLD, and CDC guidelines all advocate for screening with HBsAg; there is no consensus on the use of additional screening markers. The risk of HBV reactivation in HBsAg-negative/anti-HBc positive patients appears to be low with standard solid tumor chemotherapy[30, 31]. However, with anti-CD20 therapy, up to 24% of patients may experience HBV reactivation, including severe and even fatal cases [32]. A recent study showed that pre-emptive entecavir therapy prevented HBV reactivation in lone anti-HBc positive patients receiving rituximab-based chemotherapy but the same study also found that antiviral therapy at the time of reappearance of HBsAg or HBV DNA also prevented clinical outcomes. Therefore whether pre-emptive therapy or serial monitoring is the optimal management strategy for HBsAg-negative, anti-HBc positive patients remains unclear. Identification of lone anti-HBc positive patients is still important if prolonged or potent IST is scheduled, and therefore patients should be screened for both HBsAg and anti-HBc with a plan to implement antiviral therapy for HBsAg-positive patients and monitoring or treatment for those who are positive only for anti-HBc.

In this study, 30% of all physicians reported that they would use liver enzyme tests to screen for chronic HBV infection, a common strategy among rheumatologists and oncologists in previous studies [27, 33, 34]. Overall, 11% of patients were only tested for hepatitis B serology after an increase in liver enzymes while on IST. This is concerning because most patients with unrecognized chronic HBV infection have normal liver enzymes, and antiviral prophylaxis started at the time of ALT elevation has limited effect on the course of chemotherapy-associated HBV reactivation[35]. Compared to therapy given in response to elevated liver enzymes, prophylactic antiviral therapy has demonstrated a survival benefit, reduced the severity of both HBV reactivation and liver failure, and reduced the rate of chemotherapy interruption due to reactivation [7, 9, 36, 37]. The provision of prophylactic therapy requires that patients with chronic HBV infection are identified prior to reactivation and, therefore, prior to the elevation of liver enzymes.

Our study has some important limitations. First, physician participants were recruited from a single large academic institution. Tran and colleagues reported that community-based physicians were less likely to screen for HBV infection than physicians at academic sites[34] and thus it is possible that our results may overestimate the rate of screening for HBV prior to IST. Second, although the number of physician participants included in this study was relatively low, the majority of members from the respective divisions were included. Third, our study focused only on the country of origin as a significant risk factor for HBV. Most HBV exposures beyond childhood result in acute but not chronic HBV, leaving individuals at a very low risk of HBV reactivation with all but very potent IST. While physicians did cite other risk factors in some individuals to justify screening, the fact that screening rates were extremely low in those from HBV-endemic countries indicates that many at-risk patients were missed and if screening rates were calculated in patients with any risk factor, they would likely have been even lower than reported here.

In conclusion, this single-centre study of physicians administering IST showed that physicians did not reliably identify patients at high risk of chronic HBV infection and had low rates of screening for HBV among at-risk patients. These findings suggest that selective screening for HBV is unlikely to be a successful strategy for preventing iatrogenic HBV reactivation. Greater consistency between guidelines from different societies would reduce confusion and help to unify practice patterns across specialties. Educational campaigns to increase physicians’ knowledge of HBV risk factors could also be employed. However, based on limited success of educational strategies in other settings [25], our data support the adoption of universal screening for chronic HBV for all patients receiving IST.
